# Sino-Nasal Outcome Test-22: translation, cross-cultural adaptation, and validation in Polish-speaking patients

**DOI:** 10.1007/s00405-024-08919-z

**Published:** 2024-08-28

**Authors:** Joanna Morawska, Joanna Jeruzal-Świątecka, Piotr Politański, Wioletta Pietruszewska

**Affiliations:** 1 Department of Otolaryngology, Head and Neck Oncology, Medical University of Lodz, Łódź, Poland; 2 Department of Electromagnetic Hazards, Nofer Institute of Occupational Medicine, Łódź, Poland

**Keywords:** Chronic rhinosinusitis, CRSwNP, Sino-Nasal Outcome Test, SNOT − 22, Quality of life

## Abstract

**Purpose:**

There are many specific instruments for assessing the quality of life (QoL) in patients with chronic rhinosinusitis. Of all these tests, the Sino-Nasal Outcome Test–22 (SNOT-22) is the most widely used internationally. The purpose of the study was linguistic adaptation and validation of the SNOT-22 scale in the Polish language.

**Methods:**

The SNOT-22 was adapted into Polish and was administered to 148 subjects (108 patients with chronic rhinosinusitis with nasal polyps (CRSwNP) and 40 asymptomatic controls. Seventy-one patients completed the SNOT-22 a second time to evaluate test-retest reliability. The Polish SNOT-22 was assessed for internal consistency, test-retest reliability, discriminant validity, criterion validity, and sensitivity and specificity.

**Results:**

The Polish SNOT-22 exhibited satisfactory psychometric properties. A high Cronbach’s alpha coefficient (α = 0.89) was obtained. Significantly higher scores (*p* < 0.01) were revealed in the Study Group with a median score of 32 (range 15–53) points in comparison with controls: 5 (range 0–20). A moderate correlation was found between SNOT-22 and the Lund-Kennedy test score (*r* = 0.334; *p* < 0.001) and a strong correlation between SNOT-22 and the Lund–Macay test score (*r* = 0.469; *p* < 0.001). The best cut-off point was set at a 16 score with a sensitivity of 0.981 and a specificity of 0.995. The determined Area Under Curve (AUC = 0.997; *p* < 0.001) confirms the diagnostic accuracy of the Polish SNOT-22.

**Conclusions:**

The Polish version of the SNOT-22 is a valid and reliable tool for measuring health-related quality of life in patients with CRSwNP in the Polish-speaking population.

**Supplementary Information:**

The online version contains supplementary material available at 10.1007/s00405-024-08919-z.

## Introduction

Chronic rhinosinusitis (CRS) is one of the most commonly encountered chronic medical conditions worldwide, affecting all age groups. CRS is defined as *an inflammation of the nose and paranasal sinuses which persists for more than 3 months characterized by nasal discharge and/or obstruction*,* facial pain and/or pressure*,* and decreased sense of smell* [1, 2]. In 2012, the European Position Paper on Rhinosinusitis and Nasal Polyps (EPOS) proposed diagnostic criteria for CRS, which include the following: two or more symptoms, one of which should be either nasal blockage or nasal discharge, ± facial pain/pressure, ± hyposmia or anosmia; and either endoscopic signs of polyps and/or mucopurulent discharge from middle meatus and/ or edema/mucosal obstruction in middle meatus and/or CT findings showing mucosal changes within the ostiomeatal complex and/or sinuses. The duration of symptoms must be 12 weeks or more [3].

The estimated prevalence of CRS ranges between 5 and 16% worldwide [4–7]. The main CRS syndrome presentations include CRS with nasal polyps (CRSwNP, benign inflammatory outgrowths of the mucosa and, less commonly, allergic fungal rhinosinusitis) and CRS without nasal polyps (CRSsNP) [2].

The etiology and pathogenesis of both forms remain areas of active research. The epidemiology of CRS is modified and ultimately determined by risk factors such as genetic/hereditary, demographic, and environmental, and imparted by predictive pre-/comorbid disease [8].

The condition is diagnosed based on symptoms, endoscopic examinations, and CT changes. The choice of CRS treatment is influenced by many factors including past medication, duration of symptoms, and the presence of allergy/nasal polyps. Functional endoscopic sinus surgery (FESS) has become an effective treatment for CRS that cannot be controlled with conservative treatment [9]. Recent double-blinded clinical trials have demonstrated the efficacy of biological treatment for severe uncontrolled chronic rhinosinusitis with nasal polyps (CRSwNP) [10, 11].

Chronic rhinosinusitis negatively affects the quality of life (QOL), therefore, determining the impact of CRS on patient quality of life is an important starting point for treatment decisions, and is also critical for longitudinal assessment of response to particular treatments [12]. There have been multiple CRS-specific patient-reported outcome measures proposed over the past 20 years, but the 22-item Sinonasal Outcome Test (SNOT-22) is the most widely used instrument [13].

The questionnaire was initially developed and psychometrically validated in the English language [14] and proved superior to 14 other QoL questionnaires for the evaluation of patients with CRS due to its reliability, validity, responsiveness, and ease of use. SNOT-22 covers 22 symptoms reflecting the health burden of the rhinological patient (©2006, Washington University, St. Louis, MO). Some of the symptoms are caused by the changes at the level of the nasal cavity – nasal obstruction, posterior rhinorrhea, purulent rhinorrhea, the constant need to blow one’s nose, anosmia, and sneezing. Others refer to auricular damage, general illness, and the patient’s quality of life. Several ways in which chronic rhinosinusitis affects the patient’s quality of life are investigated: difficulty in falling asleep, waking up during the night, feeling tired after a night’s sleep, drowsiness, low work productivity, frustration/irritability, embarrassment, and sadness. The scale ranges from 0 (perfect score—no symptoms or impact on QoL) to 110 (the highest possible score suggesting severe symptoms and major impact on QoL [12, 15].

Regional differences may cause variations in the prevalence of CRS in populations. For this reason, the SNOT-22 scores if to be used as an assessment tool should be calculated regionally for normal populations [16]. To date, SNOT-22 has been adapted and validated in several other languages [5, 15, 17–27] and is now internationally recognized as a validated scoring assessment tool in the management of CRS. Its use has also been confirmed in the 2023 EPOS/EUFOREA update on the indication and evaluation of Biologics in Chronic CRSwNP. One of the indicators for biological treatment is significantly impaired quality of life as measured by SNOT-22. The recommended cut-off is SNOT-22 ≥ 40 [28].

The current study aimed to translate, adapt, and validate the SNOT-22 questionnaire for its use by Polish-speaking patients, as well as to evaluate the psychometric properties of the translated version, following the recommendations of the international consensus-based standards for the selection of health measurement instruments.

## Material and method

### Participants

The case-control study included a total number of 148 subjects, 57 women and 91 men, aged 44.13 ± 13.80 years [mean ± SD]. All participants of the study were 18 years or older and of Caucasian origin. The Study Group consisted of 108 patients with CRSwNP, 43 women and 65 men, aged 46.39 ± 13.73 years [mean ± SD] who underwent functional endoscopic sinus surgery (FESS) for CRSwNP operated in the Department of Otolaryngology, Head and Neck Oncology, Medical University of Lodz, The Norbert Barlicki Memorial Teaching Hospital, Lodz, Poland. The Control Group included 40 asymptomatic patients, without a history of chronic or acute rhinosinusitis, who were hospitalized for other reasons. A nasopharyngeal endoscopic examination was performed to confirm the non-existence of any rhinological disease in the Control Group.

The exclusion criteria for both groups included known autoimmune dysfunction, immune deficiency, primary ciliary dyskinesia, cystic fibrosis, any history of radiation exposure to the paranasal sinuses, and oncological treatment.

Data collection took place between 2018 and 2023. Approval for this study was granted by the Bioethics Committee of the Medical University of Lodz (decision no. RNN/05/18/KE). All patients signed voluntary, informed consent to this study. Confidentiality was ensured by a numerical cross-referencing system.

### Procedure

#### Laryngological examination

A demographic and clinical data questionnaire concerning information such as age, height, weight, and additional information about asthma, chronic obstructive pulmonary disease (COPD), and allergy.

For the objective evaluation, the severity of the disease in endoscopic nasal examination and the computed tomography of the paranasal sinuses was analyzed according to the Lund-Kennedy and Lund-Mackay scales.

Endoscopic examination of the nasal cavities was graded in a 3-point classification system (0-absence of polyps; 1-polyps in middle meatus only; 2-polyps beyond middle meatus but not blocking the nose completely; 3-polyps completely obstructing the nose) (Lund & Kennedy, 1997). The Lund-Mackay score [29] was used to stage the computed tomography (CT) scans conducted before the surgery. Scoring for all sinus systems, except the ostiomeatal complex was as follows: 0 = no abnormalities, 1 = partial opacification, 2 = total opacification. For the ostiomeatal complex: 0 = not occluded, 2 = occluded. A total score of 0 to 24 is possible, and each side can be considered separately (0 to 12). A mild disease is defined as a CT score of 0–7 inclusive, moderate from 8 to 15, and severe from 16 to 24.

#### SNOT-22 translation and adaptation procedure

The procedures of translation and adaptation to Polish were conducted following proposed guidelines that involved translation, synthesis of translations, back translation, committee review, and testing of the pre-final version [30].

##### Translation procedure

The forward translation was performed by the first author of the current study, (i.e., the informed translation) and, independently, by a translator blind to the purpose of the study, (i.e., the uninformed translation). Following this, the research team discussed the discrepancies between the two forward translations and combined them into a synthesized Polish version. Subsequently, a native English speaker fluent in Polish who had not participated in the prior stage of the translation completed the back translation of this version. A panel of experts, ENT specialists, reviewed the translation for the best possible phrasing. After reviewing the back translation only some minor semantic changes and modifications were made.

##### Pilot study

**Fig. 1:**
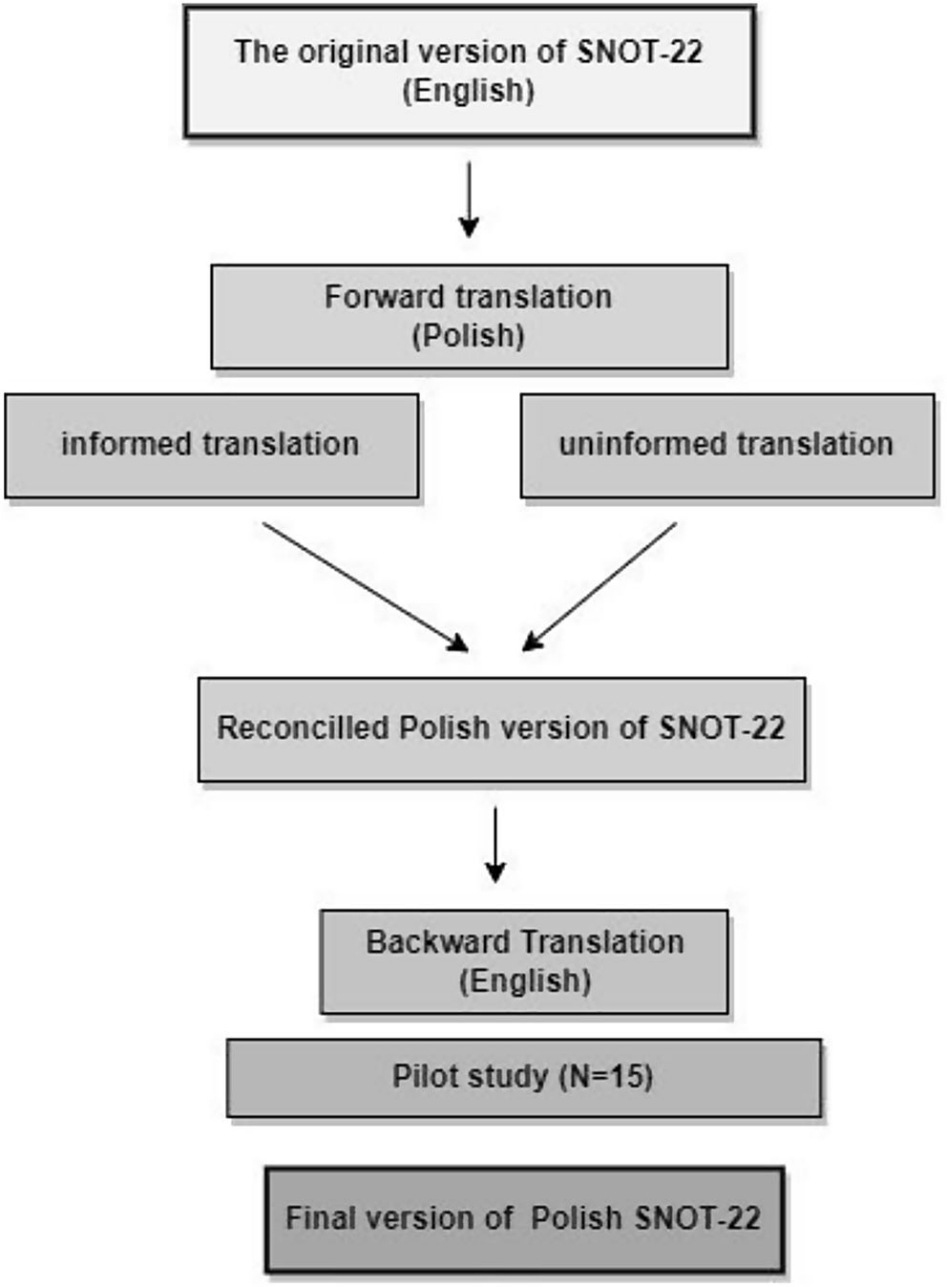
A flowchart of the SNOT-22 translation protocol

The pre-test version of the Polish SNOT-22 was administered to 15 participants from the target setting to assess comprehensibility, readability, and typographical accuracy to minimize misunderstanding and subsequent measurement errors. Each person completed the questionnaire and was interviewed for content and response for each item. Both the meaning of the items and responses were discussed by the author (JJŚ) with each completing participant. None of the respondents reported that they had had difficulties understanding the questions. No changes were made to the questions resulting in the final version of the Polish SNOT-22. (Appendix 1) The steps of the translation protocol are shown in Fig. 1.

### Statistical analysis

Statistical analysis was performed by means of IBM SPSS Statistics version 20. The results were considered statistically significant if the p-value was less than 0.05.

Due to the lack of normal distribution of analysed data (only participants’ age was distributed normally) medians and ranges were calculated for continuous variables and frequencies and percentages for categorical variables. For informative purposes, as most papers present means and standard deviations, we decided to show also those parameters.

#### Test-retest reliability

Test-retest correlation is a measure of reliability obtained by administering the same test twice over a period of time to a group of individuals. It provides an indication of stability over time. To determine test-retest reliability the questionnaire was administered among 71 participants of the Study Group within a 7 to 10-day interval. This time interval was considered short enough to avoid substantial symptom changes and long enough for the participants to recall their previous responses. Patients with any change in conservative treatment after completing the first questionnaire (medication, nasal steroids, other) or change of symptoms due to upper or lower airway infections were not included. The reproducibility was investigated by calculating the intra-class correlation coefficient (ICC), in a 2-way random model for agreement, between the test and re-test. Additionally, differences between the test and retest answers for each questionnaire item were analysed using the Wilcoxon signed-rank test.

#### Internal consistency

Internal consistency reliability shows the coherence between the items of the questionnaire and is estimated by calculating Cronbach´s alpha. Individual items’ contribution to the reliability index was assessed. Values higher than 0.70 are considered indicators of good internal consistency, using a sample big enough of 30–40 patients to prove it [31].

#### Floor and ceiling effects

The presence of floor or ceiling effects may have a negative impact on the quality of the instrument in that the psychometric properties of the questionnaire might be falsified. A component was considered to demonstrate a floor or ceiling effect if more than 15% of participants scored the minimum (floor) or the maximum possible score (ceiling) leading to implications on the questionnaire’s reproducibility and responsiveness.

#### Discriminant validity

The mean values of the Polish SNOT 22 in the Study Group and Control Group were compared for discriminant validity using the Mann-Whitney U test.

#### Construct validity

To deal with the parametric and non-parametric variables of the study, Spearman rank correlation (rho) was performed to investigate the correlation between SNOT-22 mean scores and the scores of the Lund-Kennedy scale and Lund-Mackay scale scores.

#### Sensitivity and specificity

A ROC curve was constructed for the total score of SNOT-22 to assess the test’s ability to discriminate the subjects with and without CRSwNP, as well as for individual items of the scale to determine the discriminant power of each question. The area under the ROC curve (AUC) classifies the level of accuracy of a diagnostic test. An area of 1 represents a perfect test; an area of 0.5 represents a diagnostic fail - a test that does not predict outcomes very well.

AUC values above 0.80 are generally considered clinically useful, while values below 0.80 are considered of limited clinical utility [32].

## Results

### Participants

The Study Group included 108 patients with CRSwP, 43 women and 65 men, mean age 46.4 ± 13.7 [mean ± SD]. The median age of the patients was 47.5 ranged 19–74 years. The Control Group consisted of 40 healthy subjects 14 women and 26 men, mean age 38.03 ± 12.16 [mean ± SD]. The median age in the Control group was 40 years ranged 18–71 years. The Study Group and Control Group did not differ significantly in terms of height, weight, and Body Mass Index (BMI (*p* > 0.57). In the Study Group, 19.44% (*N* = 21) of the examined patients reported asthma. Regarding allergies, seasonal allergies were reported by 21.3%(*N* = 23) and year-round allergies by 26.85% (*N* = 29) of Study Group patients. Detailed characteristics of the study population are presented in Table 1.

**Table 1:** Characteristics of the Study Group (*N* = 108) and control group (*N* = 40)

		Study group (*N* = 10*8*)	Control group (*N* = 40)	
Gender N (%)	F	43 (39.81%)	14 (35%)	*p* = 0.368
	M	65 (60.19%)	26 (65%)	
Age	mean (SD)	46.39 (13.73)	38.03 (12.16)	*p* = 0.001
	median	47.5	40	
	min	19	18	
	max	74	71	
Weight	mean (SD)	81.29 (17.15)	78.95 (11.8)	*p* = 0.576
	median	83.5	78.5	
	min	45	58	
	max	127	105	
Height	mean (SD)	172.89 (9.23)	172.6 (9.76)	*p* = 0.832
	median	173.5	172	
	min	153	153	
	max	195	191	
BMI	mean (SD)	27.12 (4.97)	26.63 (4.41)	*p* = 0.598
	median	26.42	26.01	
	min	16.53	20.56	
	max	39.45	36.74	
Asthma (%)		21 (19.44%)	2 (5%)	
Seasonal allergies (%)		23 (21.3%)	1 (2.5%)	
Year-round allergies (%)		29 (26.85%)	3 (7.5%)	
Tabaco usage (%)		16 (14.81%)	12 (30%)	

F = female, M = male, BMI = body mass index, SD = standard deviation

The scoring of the diagnostic nasal endoscopy findings in the Study Group was based on the Lund Kennedy endoscopic grading system. The median total score was 2 (range 0–6) which is indicative of the polyps beyond the middle meatus but not blocking the nose completely. The median total score for the Lund-Mackay scale was 9 (range 1–24) indicating moderate disease according to the computed tomography (CT) scans. The results of the scoring of the nasal endoscopy and the CT scans are summarized in Table 2.

**Table 2:** Lund-Kennedy and Lund-Mackay results for Study Group (*N* = 108)

	mean	SD	SEM	MED	Min	Max
Lund-Kennedy Scale	2.48	1.29	0.12	2	0	6
Lund-Mackay Scale	9.74	5.07	0.49	9	1	24

### Test-retest reliability

For test-retest reliability, 85 patients from the Study Group were asked to complete the SNOT-22 for the second time. 10 patients did not return the questionnaire and 4 were excluded from the analysis due to incomplete questionnaires. The number of valid responses was 71 resulting in a response rate of 87%.

The Intraclass Correlation Coefficient (ICC) value obtained for the test-retest was 0.977 (95% confidence interval, lower band: 0.963; upper band: 0.985) for the Polish version of SNOT-22, indicating that the instrument has a good level of reproducibility (Table 3).

Detailed response distribution values (percentage) for each SNOT-22 item for test and re-test are presented in Table 4. There were no differences between responses in the Wilcoxon signed-rank test.

**Table 3:** Test-retest reliability (Intraclass correlation [ICC]) of the Polish SNOT-22

SNOT-22 items	ICC	95% Confidence Interval	
		Lower band	Upper band
Total	0.977	0.963	,985
1	0.885	0.821	0.927
2	0.926	0.884	0.953
3	0.992	0.988	0.995
4	0.966	0.945	0.979
5	0.871	0.800	0.917
6	0.920	0.874	0.950
7	0.897	0.840	0.935
8	0.974	0.958	0.984
9	0.951	0.922	0.969
10	0.890	0.829	0.930
11	0.940	0.905	0.962
12	0.973	0.957	0.983
13	0.964	0.943	0.978
14	0.966	0.946	0.978
15	0.915	0.876	0.946
16	0.915	0.866	0.947
17	0.919	0.873	0.949
18	0.960	0.937	0.975
19	0.921	0.875	0.950
20	0.920	0.876	0.950
21	0.954	0.927	0.971
22	0.863	0.790	0.913

**Table 4:** Response distribution for each SNOT-22 item in Time Point 1 and Time Point 2

Item	Response category %					
	No Problem	Very Mild Problem	Mild or Slight Problem	Moderate Problem	Severe Problem	Problem as bad as it can be
1 Timepoint 1 Timepoint 2	0 0	2.8 2.8	5.6 8.5	50.7 49.3	40.8 35.6	0 0
2 Timepoint 1 Timepoint 2	50.7 50.7	33.8 31.0	15.5 18.3	0 0	0 0	0 0
3 Timepoint 1 Timepoint 2	49.3 49.3	11.3 9.9	16.9 16.9	16.9 18.3	5.6 5.6	0 0
4 Timepoint 1 Timepoint 2	54.9 56.3	11.3 8.5	22.5 18.3	9.9 14.1	1.4 2.8	0 0
5 Timepoint 1 Timepoint 2	0 0	0 0	12.7 16.9	39.4 35.2	39.4 35.2	8.5 12.7
6 Timepoint 1 Timepoint 2	1.4 1.4	0 0	36.6 29.6	22.5 29.6	31.0 28.2	8.5 11.3
7 Timepoint 1 Timepoint 2	66.2 67.6	32.4 29.6	1.4 2.8	0 0	0 0	0 0
8 Timepoint 1 Timepoint 2	74.6 71.8	0 4.2	12.7 12.7	11.3 8.5	1.4 2.8	0 0
9 Timepoint 1 Timepoint 2	90.1 88.7	4.2 5.6	5.6 4.2	0 1.4	0 0	0 0
10 Timepoint 1 Timepoint 2	1.4 4.2	38.0 32.4	38.0 42.3	22.5 18.3	0 2.8	0 0
11 Timepoint 1 Timepoint 2	21.0 21.0	5.6 8.5	39.4 36.6	28.2 25.4	5.6 7.0	0 1.4
12 Timepoint 1 Timepoint 2	19.7 18.3	7.0 12.7	32.4 29.6	32.4 29.6	8.5 9.9	0 0
13 Timepoint 1 Timepoint 2	21.1 23.9	5.6 5.6	23.9 18.3	32.4 35.2	16.9 16.9	0 0
14 Timepoint 1 Timepoint 2	19.7 21.1	5.6 2.8	15.5 16.9	35.2 35.2	23.9 23.9	0 0
15 Timepoint 1 Timepoint 2	7.0 5.6	26.8 37.5	36.6 27.1	26.8 26.8	2.8 2.8	0 1.4
16 Timepoint 1 Timepoint 2	69.0 67.6	21.1 18.3	8.5 11.3	1.4 2.8	0 0	0 0
17 Timepoint 1 Timepoint 2	59.2 62.0	26.8 22.5	14.1 12.7	0 2.8	0 0	0 0
18 Timepoint 1 Timepoint 2	36.6 45.1	33.8 25.4	12.1 11.3	7.0 8.5	9.9 8.5	0 1.4
19 Timepoint 1 Timepoint 2	38.0 46.5	46.5 36.6	12.7 14.1	2.8 2.8	0 0	0 0
20 Timepoint 1 Timepoint 2	31.0 32.4	36.6 31.0	22.5 25.4	9.9 11.3	0 0	0 0
21 Timepoint 1 Timepoint 2	4.2 5.6	16.9 16.9	26.8 23.9	26.8 28.2	18.3 18.3	7.0 7.0
22 Timepoint 1 Timepoint 2	1.4 1.4	0 1.4	31.0 26.8	42.3 43.7	23.9 23.9	1.4 2.8

### Internal consistency

The measurement of the internal consistency showed a very high Cronbach’s alpha coefficient for the total SNOT-22 score: α = 0.89. A detailed analysis of each item of the test indicates that Cronbach’s alpha value did not increase significantly after eliminating any item, meaning that all of the items have a comparable influence on the reliability of the whole scale (Table 5).

**Table 5:** Internal consistency (Cronbach’s alpha) of the Polish SNOT-22 − item analyses

Snot-22 Item #	Cronbach’s alpha if item deleted
1	0.876
2	0.892
3	0.889
4	0.888
5	0.877
6	0.876
7	0.890
8	0.888
9	0.892
10	0.879
11	0.882
12	0.882
13	0.882
14	0.880
15	0.881
16	0.894
17	0.890
18	0.888
19	0.889
20	0.885
21	0.881
22	0.881

### Floor and ceiling effects

**Fig. 2:**
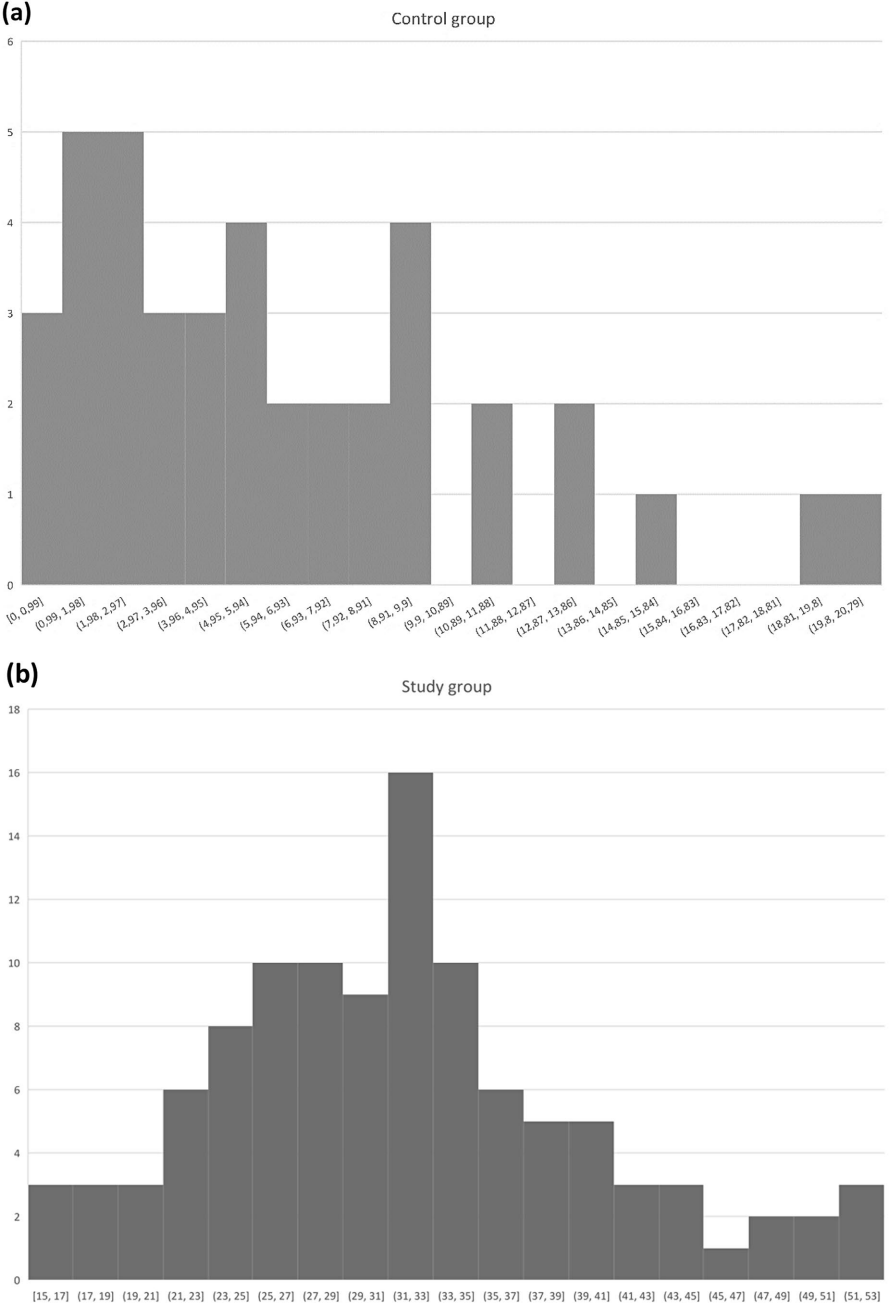
SNOT-22 Total score frequency distribution in (**a**) Control Group (*N* = 40) and (**b**) Study Group (*N* = 108)

There was no floor and ceiling effect for the Polish SNOT-22 test scores in the Study Group and Control Group. The total score frequency distribution in both groups is presented in Fig. 2.

### Discriminant validity

The results of the SNOT-22 in the Study Group and Control Group differ significantly (*p* < 0.001) (Table 6). The median SNOT-22 score in the Study Group was significantly higher than in the Control Group with 32 (range 15–53) points and 5 (range 0–20) points respectively for the Study Group and Control Group. Mann-Whitney U test showed that this difference was statistically significant (*p* < 0.001). Figure 2 shows the SNOT-22 Total score frequency distribution in the Control Group and Study Group.

The problems that were rated as the most severe by the subjects of the study were #5 – *post-nasal drip* with a median score of 3 (range 1–5), #1- *need to blow nose* with 3 (range 0–4), #22 – *nose blockage* with 3 (range 0–5), and #6 – *thick nasal discharge* with 2 (range 0–5). The lowest median values were recorded for items #9 – *ear pain*: 0 (range 0–2) and #7 – *ear fullness*: 0 (range 0–2).

**Table 6:** Mean Total SNOT-22 results for Study Group (*N* = 108) and Control Group (*N* = 40)

SNOT-22 Total Score	*N*	mean	SD	MED	SEM	Min	Max	Significance
Control Group	40	5.90	5.05	5	0.79	0	20	*p* < 0.001
Study Group	108	32.08	8.34	32	1.14	15	53	

### Construct validity

A statistically significant moderate, positive correlation was observed for the Polish SNOT-22 and Lund-Kennedy scale (*r* = 0.334; p *<* 0.01). We found a statistically significant, strong positive correlation between the Polish SNOT-22 and the Lund Mackey score (*r* = 0.469; p *<* 0.01).

### Diagnostic reliability

**Fig. 3:**
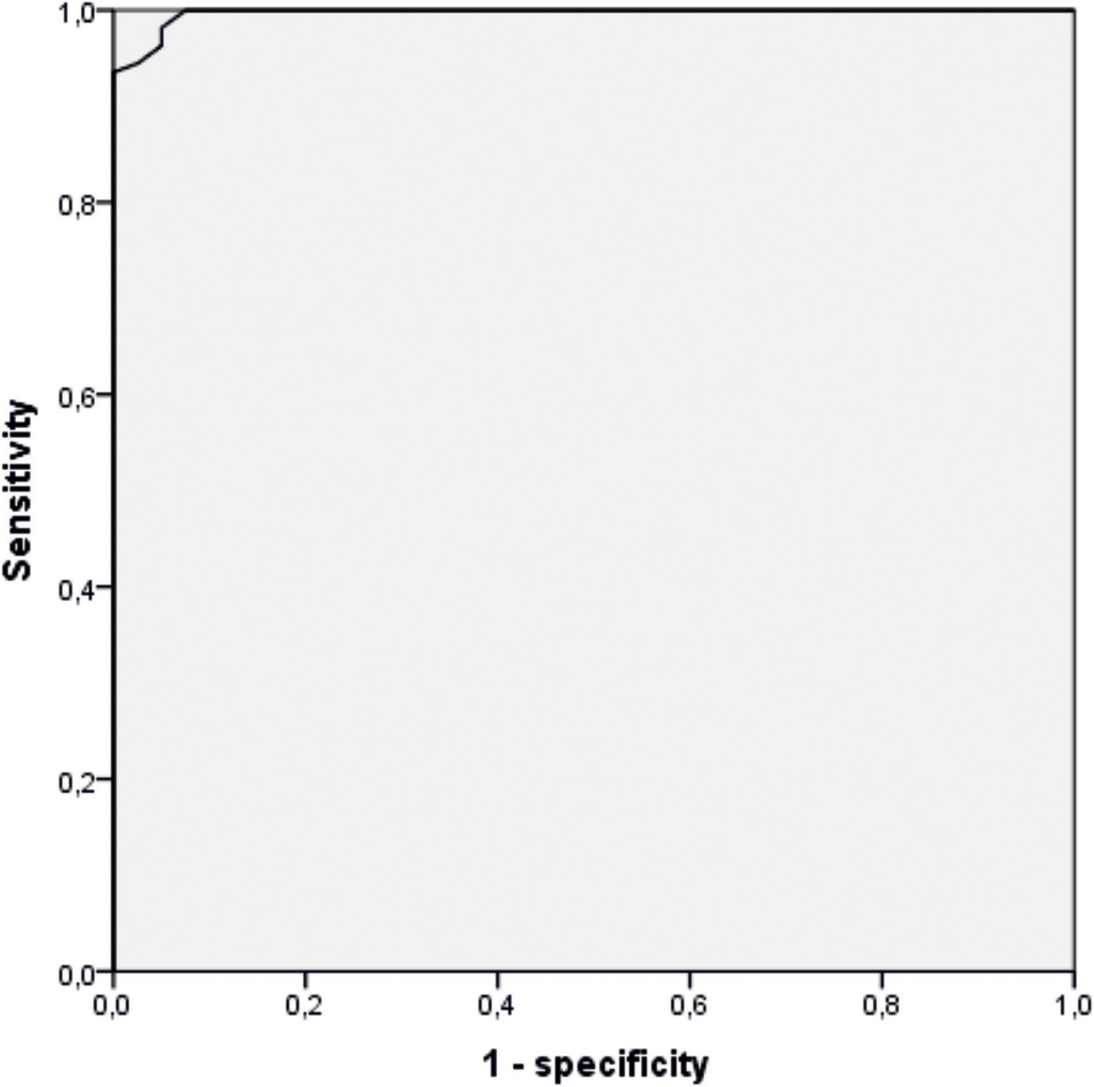
ROC curve for the Polish SNOT-22 scale

The Receiver Operating Characteristic (ROC) curve with Area Under Curve (AUC) was constructed for the diagnostic evaluation of SNOT-22. The best cut-off point that maximizes the screening value of SNOT-22 giving the best relationship between sensitivity and specificity was set at 16 value. This point with a sensitivity of 0.981 and specificity of 0.950 shows the test’s best ability to discriminate subjects with and without CRSwNP (Fig. 3).

The analysis of the determined AUC (Table 7) showed that the accuracy of SNOT-22 as a diagnostic test is excellent: AUC = 0.997; *p* < 0.001. From among 22 variables of the SNOT-22 scale, the best discriminating power (the highest value of AUC) is observed for item #6 -Thick nasal discharge: AUC = 0.973; *p* < 0.001 followed by item #1 -*Need to blow nose*: AUC = 0.967; *p* < 0.001 and item #10- *Facial pain/pressure*: AUC = 0.949; *p* < 0.001. The area under the curve is equal to or higher than 0.7 for 14 Out of 22 variables, which indicates that the questions correctly differentiate between patients with and without CRSwNP. (Table 7)

**Table 7:** Area under the curve (AUC) for the Polish SNOT-22 scale’s items and total score

SNOT-22 Item #	AUC	95% Confidence Interval	
		Lower band	Upper band
1.	0.967	0.942	0.991
2.	0.562	0.453	0.670
3.	0.670	0.582	0.758
4.	0.700	0.615	0.784
5.	0.931	0.883	0.979
6.	0.973	0.938	1.000
7.	0.671	0.585	0.758
8.	0.577	0.479	0.675
9.	0.551	0.451	0.651
10.	0.949	0.901	0.997
11.	0.774	0.694	0.855
12.	0.759	0.677	0.841
13.	0.757	0.673	0.842
14.	0.788	0.706	0.871
15.	0.944	0.908	0.981
16.	0.539	0.431	0.647
17.	0.694	0.611	0.778
18.	0.709	0.618	0.801
19.	0.787	0.717	0.857
20.	0.806	0.738	0.873
21.	0.885	0.819	0.950
22.	0.897	0.822	0.972
Total	0.997	0.993	1.000

## Discussion

This work aims to describe the translation, cross-cultural adaptation, and validation process of the Polish version of SNOT-22. Reliability, validity, and responsiveness are specific characteristics of each context, so an instrument that has demonstrated satisfactory psychometric properties in a specific population is not necessarily appropriate for others. Thus, the validation of the Polish SNOT-22 will allow more specific treatment for CRS patients, as well as its generalization in the scientific community, and comparison between different countries. Among numerous disease-specific sinonasal outcome questionnaires the SNOT-22 questionnaire showed reliability, validity, responsiveness, and ease of use [33]. This assumes particular importance in the era of the introduction of biological treatment in CRSwNP patients with or without asthma (omalizumab, benralizumab, dupilumab). At present, the SNOT22 is widely used to assess the quality of life of these patients and it will be possible to compare results between patients of different nationalities, provided validated tools are used.

The number of participants in international SNOT-22 validation studies varied greatly. The largest group was that in the original validation study by Hopkins et al. [14] a prospective cohort study collecting data on 3128 adult patients undergoing sinonasal surgery in 87 NHS hospitals in England and Wales. In some studies on validation of the SNOT-22 the total number of subjects was under one hundred [34, 35]. In other studies, this number exceeded two hundred participants, for instance, 206 in Brazilian Portuguese validation [19], 341 in Italian [22], and 422 in French [20] studies. A total number of 148 enrolled subjects places our study at average in terms of sample size.

The participants in our study were all diagnosed with chronic rhinosinusitis with nasal polyps (CRSwNP). In other studies, the study sample included patients with CRS with and without nasal polyps.

Validation studies determine the accuracy, dependability, and consistency of a tool. The Polish SNOT-22 exhibited satisfactory psychometric properties. The results demonstrate that the Polish version of the SNOT-22 is a reliable outcome measure according to 2 reliability tests: internal consistency and test-retest reproducibility. The Cronbach’s alpha coefficient α = 0.89 showed similar values in the Polish SNOT-22 as compared with the original English version α = 0.91 [14] and compared with translations of the questionnaire to other languages. [15, 23–27, 36] Test-retest reproducibility of the Polish SNOT-22 was determined by the Intraclass Correlation Coefficient (ICC). The ICC value obtained for the test-retest resulting in 0.977 (95% confidence interval, lower band: 0.963; upper band: 0.985) for the Polish version of SNOT-22 indicates excellent reliability and stability over time, given the ICC can take a value from 0 to 1, with 0 indicating no agreement and 1 indicating perfect agreement. This result is in line with those obtained in other international SNOT-22 validation studies [17, 18].

The ability of the questionnaire to distinguish the disease-affected group was tested by comparison with asymptomatic subjects. Mean total SNOT-22 scores reported in different SNOT-22 translations range from 25.6 ± 13.3 (mean ± SD) in Persian translation [37], 29.7 (range 7–67) in Danish translation [38] to 62.4 ± 7.9 (mean ± SD) in Arabic [35] and 62.4 ± 25.3 (mean ± SD) in Brazilian Portuguese translation [23]. Our clinical sample of CRSwNP patients had a mean SNOT-22 score of 32.08 ± 8.34 (mean ± SD) which is in the lower range of the results reported in other studies. Several factors result in the discrepancy between the mean total scores reported in the literature, such as the demographic characteristics of the groups, or the patient recruitment methods [25]. It should also be taken into account that more than half of SNOT-22 domains are non-rhinology specific, therefore the scores can be influenced by the presence of various related conditions such, but not exclusively, as asthma, allergic rhinitis, chronic throat symptoms, Eustachian tube dysfunction, ear pathologies, sleep disturbances, depression, and also socioeconomic factors affecting QoL [39].

In 2020 Gallo et al. conducted a study aimed at verifying in an Italian CRS population whether SNOT-22 could assist physicians in predicting surgical outcomes, improving the shared decision-making process, and ameliorating patients’ understanding of their QoL expectations after treatment [40]. Based on the baseline SNOT-22 score, the cohort of patients was divided into 10 groups. The primary outcomes included measurement of the percentage of patients receiving a minimal clinically important difference (MCID) and the percentage of relative improvement (RI) after surgical treatment. Based on the results of the study, the mean percentage of achieving an MCID in groups 3–10 (patients with mean SNOT-22 scores of 30–110 points) is 91.6% with an average of 56.8% of RI. Contrarily, the mean percentage of achieving an MCID in groups 1–2 (patients SNOT-22 mean scores of 10–29) is 44.2% with an average of 38.9% of RI. A similar conclusion was drawn by Farhood et al. who conducted a cross-sectional study and systematic review on SNOT-22 in a control population. The authors underlined that in CRS patients with preoperative scores < 20, FESS is unlikely to yield clinical improvement [41]. In light of these data, the classification of patients with CRSwNP for FESS in our study was justified, as they were likely to benefit from surgery, with the SNOT-22 median score of 32 points standing a greater than 75% chance of achieving an MCID and on average obtain a 45% relative improvement in their QoL after ESS.

When validating a tool for use in the clinical setting It is necessary to establish its ‘normal’ value within the general population to set a reference point to identify those who may benefit from treatment. Gillet et al. [42] conducted a pilot study of the SNOT-22 score in adults with no sinonasal disease to determine a normal SNOT-22 score. Based on the results of a total of 116 subjects they concluded that in a clinical situation, a SNOT 22 score of 7 should be used as a guide for ‘‘normal’’, and that care should be taken when suggesting treatment on patients with a score below this level. The group of healthy controls in our study had a median total SNOT-22 score of 5 (range 0–20), and although slightly lower, it is in accordance with the result of the aforementioned study. In other studies validating the SNOT-22 the results in the control group were much higher. We found only one study with the controls’ result lower than the suggested reference point of 7 and it was the study by de los Santos et al. where the median SNOT-22 value for controls was 2 points [26]. In the remaining studies, the mean results in controls ranged from 8.7 ± 8.1 [mean ± SD] to 19.5 ± 13.1 [mean ± SD] [17, 18, 21–24, 27, 36].

It should be stressed at this point that despite the seemingly promising prognostic value of the SNOT-22, PROM questionnaires should not be used as a replacement for nasal endoscopy and/or CT sinus scan for diagnosis and assessment of CRSwNP [39]. Therefore, although the baseline SNOT-22 score and the chance of achieving the MCID are not intended to be used as an absolute threshold for eligibility for surgery, results reported in international studies suggest that a patient with a low preoperative score might be less likely to benefit from surgery and caution should be paid when operating on patients with a score < 10 [40].

When conducting the literature research on the SNOT-22 adaptation and validation in different languages, we found only a few studies that undertook the subject of diagnostic reliability of the SNOT-22 as determined by the Receiver Operating Characteristic (ROC) curve with Area Under Curve (AUC). In the validation study of the Turkish SNOT-22 Cakir et al. reported that with the cut-off value of 33.5, the sensitivity and specificity of the Turkish version of the SNOT‐22 were 54.5% and 75.9%, respectively (95% CI, (AUC): 0.69, range 0.624–0.756, *p* = 0.000) [36]. The Arabic validation study by Alanazy et al. in the ROC curve analysis of discriminant validity with the AUC of 0.87 at a cut-off threshold of 18.5, sensitivity was 77%, and specificity was 84% [35]. In the study of Vaitkus et al. the ROC test indicated that in the Lithuanian version of the SNOT-22, the total score of 29 was the optimal score distinguishing between patients and healthy controls The sensitivity of the Lithuanian version of SNOT-22 was 91.7%, and specificity 82.6% [25]. Adnane et al. conducted psychometric validation of a Moroccan version of the SNOT-22 in which they assessed the discriminant validity using a receiver operating characteristic (ROC) curve [17]. The determined area under the curve (AUC) was 0.994. The cut-off point was not reported in this study. In our study, the AUC equaled 0.997 with *p* < 0.001 and this result was the highest among the aforementioned studies. The cut-off threshold of 16 (sensitivity = 0.981, specificity = 0.995) was the closest to that in the study by Alanazy et al. [35].

Psychometric properties such as sensitivity and specificity are common measures used to evaluate the quality of a screening test. These psychometric properties are unbiased if the screening test results are compared with a gold standard measure. In our studies two objective methods of chronic rhinosinusitis were applied: endoscopic examination of the nasal cavities graded according to the Lund-Kennedy scale and computed tomography (CT) scans graded according to the Lund-Mackey scale.

Nasal endoscopy plays a main role in recognizing anatomical structural variations and mucosal changes of the middle meatus and osteomeatal complex. Adnane et al. reported a good statistically significant correlation between the SNOT-22 scores and the Lund-Kennedy endoscopic scores (*r* = 0.71) [17]. In our study, the correlation was also statistically significant, but moderate (*r* = 0.334). Other studies we found did not report a correlation between these two measures.

A computed tomography scan of the paranasal sinuses is regarded as the gold standard diagnostic radiological tool for CRS. Although it is widely used in the assessment of chronic rhinosinusitis [29] disagreement exists about the relationship between Lund-Mackay CT scores and quality-of-life outcome measures. In 2018 Brooks et al. [43]. reported that the preoperative Lund-Mackay scale scores were significantly associated with preoperative SNOT-22 scores (*p* < 0.01). Our study confirms this finding with a statistically significant, strong positive correlation between the Polish SNOT-22 and the Lund Mackey score (*r* = 0.469; p *<* 0.01). This result should, however, be interpreted with caution, as our patient sample was limited to those diagnosed with CRSwNP. In a study conducted by Bradley and Kountakis [44] in 2005, the results showed that the severity of rhinosinusitis based on a CT scan before surgery was not related to the severity of symptoms based on the SNOT-22 questionnaire after ESS surgery. Also, a CT scan could not predict the improvement of symptoms after FESS. Similarly, most of the available studies report no significant correlation between the SNOT-22 and the Lund-Mackay scores [18, 20].

The following study limitations are worth mentioning. First, the study population is limited to a sample selected for FESS. CRS patients without planned surgery were not included. However, these limitations could mostly affect the scores rather than the psychometric properties cc of the adapted version. It has been well documented that the SNOT-22 questionnaire is capable of being used as a before-after questionnaire in patients undergoing sinonasal surgeries. Our validation study is based on preoperative SNOT-22 results only. Therefore, our study does not provide information on SNOT-22 responsiveness that is the ability of the questionnaire to detect clinical changes. Future studies should focus on documenting the Polish SNOT-22’s ability to detect changes over time by comparing the final scores before and after the surgical intervention and other forms of CRS treatment. Another notion for future exploration might be an interaction of the SNOT-22 score with allergy, asthma, or smoking We did not show this interaction in the presented research, which may have resulted in a certain degree of a response bias.

## Conclusion

The analyses conducted to evaluate the psychometric properties of the Polish SNOT-22 questionnaire indicate that it is a valid tool for measuring health-related quality of life in patients with CRSwNP in the Polish-speaking population. It seems a valuable addition to the diagnostic battery of tools used in clinical practice.

## Electronic supplementary material

Below is the link to the electronic supplementary material.

Supplementary Material 1
