# Supplementary material for: Sino-Nasal Outcome Test-22: translation, cross-cultural adaptation, and validation in Polish-speaking patients
Source: Eur Arch Otorhinolaryngol. 2024 Aug 28;281(12):6431–43. doi: 10.1007/s00405-024-08919-z (PMC11564299; doi:10.1007/s00405-024-08919-z)
Supplement: Supplementary file 1 — Supplementary Material 1 [file 405_2024_8919_MOESM1_ESM.docx]

**Appendix 1**

**Kwestionariusz objawów zatokowo-nosowych (Sino-Nasal Outcome Test SNOT-22)**

Poniżej znajdzie Pan/Pani listę objawów. konsekwencji socjologicznych oraz emocjonalnych dotyczących Pana/Pani choroby zatok. Chcielibyśmy dowiedzieć się więcej na temat tych problemów i bylibyśmy bardzo wdzięczni za udzielenie odpowiedzi na poniższe pytania najszczegółowiej jak Pan/Pani potrafi. Nie ma tutaj dobrych i złych odpowiedzi. Prosimy o ocenę poszczególnych problemów tak jak Pan/Pani odczuwał/a ja przez ostatnie 2 tygodnie. Bardzo dziękujemy za wypełnienie poniższego kwestionariusza.

| 1. Biorąc pod uwagę. jak poważny jest problem. gdy go Pan/Pani doświadcza i jak często się pojawia. proszę ocenić każdy element poniżej zakreślając liczbę. która odpowiada Pana/Pani odczuciu. używając tej skali | Brak problemu | Bardzo niewielki problem | Łagodny lub nieznaczny problem | Umiarkowany problem | Poważny problem | Najgorzej jak tylko może być |  | 5 najważniejszych problemów |
| --- | --- | --- | --- | --- | --- | --- | --- | --- |
| 1. Potrzeba wydmuchania nosa | 0 | 1 | 2 | 3 | 4 | 5 |  | O |
| 1. Kichanie | 0 | 1 | 2 | 3 | 4 | 5 |  | O |
| 1. Wodnisty katar | 0 | 1 | 2 | 3 | 4 | 5 |  | O |
| 1. Kaszel | 0 | 1 | 2 | 3 | 4 | 5 |  | O |
| 1. Spływanie wydzieliny po tylnej ścianie gardła | 0 | 1 | 2 | 3 | 4 | 5 |  | O |
| 1. Gęsty katar | 0 | 1 | 2 | 3 | 4 | 5 |  | O |
| 1. Uczucie pełności w uszach | 0 | 1 | 2 | 3 | 4 | 5 |  | O |
| 1. Zawroty głowy | 0 | 1 | 2 | 3 | 4 | 5 |  | O |
| 1. Ból uszu/uczucie zwiększonego ciśnienia w uszach | 0 | 1 | 2 | 3 | 4 | 5 |  | O |
| 1. Ból lub uczucie ucisku w twarzy | 0 | 1 | 2 | 3 | 4 | 5 |  | O |
| 1. Problem z zasypianiem | 0 | 1 | 2 | 3 | 4 | 5 |  | O |
| 1. Wybudzanie się w nocy | 0 | 1 | 2 | 3 | 4 | 5 |  | O |
| 1. Niewysypianie się w nocy | 0 | 1 | 2 | 3 | 4 | 5 |  | O |
| 1. Uczucie zmęczenia po przebudzeniu | 0 | 1 | 2 | 3 | 4 | 5 |  | O |
| 1. Zmęczenie | 0 | 1 | 2 | 3 | 4 | 5 |  | O |
| 1. Obniżona produktywność | 0 | 1 | 2 | 3 | 4 | 5 |  | O |
| 1. Obniżona koncentracja | 0 | 1 | 2 | 3 | 4 | 5 |  | O |
| 1. Sfrustrowanie/ nerwowość/ rozdrażnienie | 0 | 1 | 2 | 3 | 4 | 5 |  | O |
| 1. Smutek | 0 | 1 | 2 | 3 | 4 | 5 |  | O |
| 1. Zażenowanie/ zakłopotanie | 0 | 1 | 2 | 3 | 4 | 5 |  | O |
| 1. Poczucie węchu/smaku | 0 | 1 | 2 | 3 | 4 | 5 |  | O |
| 1. Uczucie blokady/ zatkanego nosa | 0 | 1 | 2 | 3 | 4 | 5 |  | O |
| SUMA |  |  |  |  |  |  |  |  |

2. Proszę zaznaczyć najważniejsze problemy wpływające na Pana/Pani zdrowie (maksymalnie 5 pozycji) _____

*All rights reserved. Copyright 2006. Washington University in St. Louis. Missouri.*

*SNOT-20 Copyright © 1996 by Jay F. Piccirillo. M.D.. Washington University School of Medicine. St. Louis. Missouri
SNOT-22 Developed from modification of SNOT-20 by National Comparative Audit of Surgery for Nasal Polyposis and Rhinosinusitis Royal College of Surgeons of England.*
